# Needs and Perceptions of Patients With Dystonia During the COVID-19 Pandemic: A Qualitative Framework Analysis of Survey Responses From Italy

**DOI:** 10.3389/fneur.2022.808433

**Published:** 2022-06-16

**Authors:** Vittorio Rispoli, Matías Eduardo Díaz Crescitelli, Francesco Cavallieri, Francesca Antonelli, Stefano Meletti, Luca Ghirotto, Franco Valzania

**Affiliations:** ^1^Neurology, Neuroscience Head Neck Department, Azienda Ospedaliero-Universitaria di Modena, Modena, Italy; ^2^Qualitative Research Unit - Azienda USL-IRCCS di Reggio Emilia, Reggio Emilia, Italy; ^3^Clinical and Experimental Medicine PhD Program, University of Modena and Reggio Emilia, Modena, Italy; ^4^Neurology Unit, Neuromotor and Rehabilitation Department, Azienda USL-IRCCS di Reggio Emilia, Reggio Emilia, Italy; ^5^Neurology Unit, Department of Biomedical, Metabolic and Neural Science, University of Modena and Reggio Emilia, Modena, Italy

**Keywords:** COVID-19, dystonia, deep brain stimulation, quality of life, botulinum neurotoxin

## Abstract

**Introduction:**

The COVID-19 pandemic and its countermeasures have created changes in both life and healthcare. With the prioritization of COVID-19-related management, the risks and experiences of patients suffering from rare conditions, such as dystonia, during the pandemic remain understudied.

**Materials and Methods:**

Using a framework analysis of a nationwide qualitative online survey, we sought to explore the perspectives of patients with dystonia on their clinical assistance and possible unmet needs during the first pandemic wave. An online survey consisting of 37 items (such as demographic characteristics, dystonia-related features, neurological service provision, therapeutic relationship with the neurologist, perceptions related to virus infection, perceptions about healthcare-related needs, work-related questions, requesting information, and seeking support during the pandemic) was carried out using both close and open-ended questions.

**Results:**

Responses from 62 participants were collected, with most of them from the red zones in Italy, where they were confined indoors. Social isolation was a relevant stressor. Motor and non-motor symptoms increased with detrimental consequences for patients' job and daily functionality. Outpatient clinics and rehabilitation sessions were temporarily shut down, and even telephone/mail support was sparse. Despite efforts, patients felt alone in dealing with dystonia.

**Conclusion:**

The first wave of the pandemic and its related restrictions had detrimental consequences for people living with dystonia, and their relevant needs remained unmet. These findings may contribute to implementing remedial healthcare provisions in this pandemic or in future pandemics.

## Introduction

Dystonia is a hyperkinetic movement disorder characterized by abnormal intermittent or sustained postures and stereotyped twisting and tremulous movements (1, 2). According to epidemiological data, the prevalence of dystonia among the general population may be underestimated (3, 4) as the condition is probably underdiagnosed (2, 3).

Dystonic syndromes consist of motor and non-motor symptoms, such as mood disorders and pain (2, 5–7). Non-motor symptoms also play a central role in determining the quality of life (QoL) and individual functionality (7–11).

Dystonia management, therefore, encompasses regular follow-ups and various strategies frequently managed in a multidisciplinary setting (2). The main treatment plan not only consists of medical [oral drugs (2), such as benzodiazepine and botulinum neurotoxin injection (BoNT) (12)] and surgical approaches [i.e., deep brain stimulation (DBS) (2, 11, 13)] but also, more recently, rehabilitation (14, 15).

As the SARS-CoV-2 pandemic soared in early 2020, Italy became one of the main hot spots in Europe, and extraordinary measures were implemented to limit the spread of the infection (16). These countermeasures mainly impacted people's interaction and movement, with the closure of educational and leisure facilities and non-essential commercial and productive activities. In addition, medical care underwent extraordinary reorganization with the primary aim of tackling SARS-COV-2. The four most affected regions, Piedmont, Lombardy, Emilia-Romagna, and Veneto (17), all in northern Italy, were defined as “red zones” (16).

Outpatient clinics in public hospitals were temporarily shut down to contain the spread of COVID-19, especially among the most vulnerable patients (18). As a result, the care for chronic neurological conditions requiring a regular and accurate follow-up suffered a backlash. In the context of dystonia management, BoNT has short-term effects on muscle hypertonia, so it must be frequently administred (19).

In Italy, BoNT intervals were increased (12, 18, 20, 21), which negatively impacted the rehabilitation process of patients with dystonia (DPs), with botulinum neurotoxin losing its effect (18). Various BoNT-related delays have been reported: 73.61 ± 26.54 days by Erro and colleagues (12) and 9 ± 2.8 months (the maximum delay registered was 19 months) by Tarantino and colleagues (18).

Similarly, COVID-19 impacted the management of DBS, a second-level treatment for DPs, which requires routine monitoring and close follow-up, given the need for periodic adjustments of stimulation parameters and checks (13, 22–24).

While most recent studies address the overall impact of COVID-19-related conditions, the experiences of DPs and other patients with rare diseases have been neglected. This study explores the perception of patients with dystonia of their condition during the first wave of the pandemic in Italy and uncovers their unmet needs.

## Materials and Methods

We applied a descriptive qualitative design utilizing an online survey, as indicated by Braun and colleagues (25). Qualitative surveys consist of a self-administered series of close- and open-ended questions. An online qualitative survey is valuable for producing accounts of participants' subjective experiences and perceptions (25), as shown in a recent article on the needs of patients with Parkinson's disease during the COVID-19 pandemic (26).

### Participant Recruitment

Participants were anonymously recruited by following an online convenience sampling technique (27). The survey was posted on social media networks and the Italian association of dystonia website, inviting the target population to complete it. At the beginning of the study, participants were provided with a written description of the study's aim and the contact details of the principal investigator (VR). Participation was voluntary.

### Survey

We planned the survey comprising 37 items covering demographic characteristics, clinical condition, neurological service provision, therapeutic relationship with the neurologist, perceptions related to virus infection and healthcare-related needs, work-related questions, and information and support requests during the pandemic. The items were decided based on the authors' expertise and emerging literature (28). The open-ended qualitative questions were designed to allow participants to express their views on their experiences, so they were not theory or literature-informed but designed as a result of consensus among authors. The survey was available online after the first phase of the lockdown (July-August 2020) and took 10–15 min to complete.

The core open-ended questions on which this paper concentrates were:

- How do you feel about the possibility of contracting the Corona virus?
- What are the needs related to dystonia, which risk not being satisfied due to the pandemic? This question was probed with the following: “What were the needs which have not been met in this pandemic period compared to previous periods?”
- Could you describe if and how your condition worsened?
- Could you describe changes related to your work situation?
- Do you feel you need more information during the pandemic?

### Data Analysis

We analyzed the demographics, dystonic features, and other information collected from close-ended questions using descriptive statistics. Participants' education was recorded according to the level of education: primary education, spanning from 6 to 13 years of age (elementary and middle schools); secondary education, from 14 to 19 years of age (high school); and tertiary education, which consisted of any form of academic program beyond 19 years of age. We reported the frequency concerning the regions of residence of participants, which fall into three main macro-regions: northern Italy (Aosta Valley, Piedmont, Liguria, Lombardy, Emilia-Romagna, Veneto, Trentino-Alto Adige/Südtirol, and Friuli-Venezia-Giulia), central Italy (Tuscany, Umbria, Marche, Lazio, and Abruzzo), and southern Italy (Campania, Molise, Apulia, Basilicata, Calabria, Sicily, and Sardinia).

Clinical dystonic features were assessed based on the myelodysplastic syndrome (MDS) classification (1) and, when possible, according to the responses in the survey where participants were asked to specify the age of onset of dystonia, the segment of the body that was involved, the known clinical diagnosis, and ongoing therapies. Each participant's list of body segments, as reported, was categorized further by body distribution, as defined by Albanese et al. (1). However, it was not feasible to investigate more detailed clinical characteristics such as temporal pattern, etiology, and associated features.

We employed the framework analysis (FA) to analyze the open-ended questions. FA is an inductive analytical approach (29) suitable for generating themes from a multidisciplinary research sample's short and numerous answers (30).

Two of the authors (LG ane MEDC) independently read and re-read the text of the first 30 responders to identify themes and sub-themes. These thematic categories shaped the initial framework, which was further discussed with the other authors (FV, FC, FA, SM, and VR) and redefined. The finalized framework was then applied to the remaining responses by the two authors (LG ane MEDC). Since the participants had the opportunity to leave any question unanswered, the overall response to each question may not score 100%.

### Ethical Considerations

The Local Ethics Committee was approached, and we were advised that formal ethical approval was not necessary as, according to Italian law, the respondents' identities and data remained anonymous, and their demographic information was collected as aggregated data only. Along with the survey, the team provided the aim of the study, confidentiality, the Italian Law on Privacy statement, and the principal investigator's contact details. When the respondents returned the survey, informed consent was assumed.

## Results

### Study Population

Sixty-two participants answered the survey. Demographics are summarized in Table 1. Of the respondents, 46 (74.2%) were women. While 16 of the 20 Italian regions were represented in the cohort, 31 participants (50%) responded from the “red zones” (Piedmont, 9.7%, Lombardy, 12.9%, Emilia-Romagna, 21%, and Veneto, 6.5%).

**Table 1:** Respondents' characteristics.

**Age at the study**	**Frequency-**
	**responders (%)**
18–30	2 (3, 2)
31–40	7 (11, 3)
41–50	15 (24, 2)
51–60	28 (45, 2)
61–70	8 (12, 9)
71–80	2 (3, 2)
**Gender**	
Female	46 (74, 2)
Male	16 (25, 8)
**Region (residency)**	
Emilia-Romagna	13 (20, 9)
Lombardy	8 (12,9)
Tuscany	8 (12, 9)
Piedmont	6 (9, 7)
Lazio	5 (8, 1)
Liguria	5 (8, 1)
Sicily	4 (6, 4)
Veneto	4 (6, 4)
Friuli-Venezia-Giulia	2 (3, 2)
Basilicata	1 (1, 6)
Calabria	1 (1, 6)
Campania	1 (1, 6)
Marche	1 (1, 6)
Apulia	1 (1, 6)
Sardinia	1 (1, 6)
Trentino-Alto Adige/Südtirol	1 (1, 6)
**Education**	
Elementary school	0 (0)
Middle School	16 (25, 8)
High school	28 (45, 2)
University	15 (24, 2)
Ph.D. or any other post-graduation program	3 (4, 8)

Details of the dystonic features are listed in Table 2. The age of onset in the cohort was mainly late adulthood, with 58.7% above the fifth decade (skewness: 0.28). As for body distribution, it was possible to classify all but one respondent because P1 did not specify any details about which body segment was involved in dystonia. Notably, 62.9% of respondents were classified as focal dystonia. Most of the respondents (80.6%) received BoNT, 9.7% received DBS, and only two were under no therapeutic regime. Significantly, more than half of the respondents (58%) had a combined therapeutic regime.

**Table 2:** Dystonic clinical features.

**Age of onset**	**Frequency -** ***n*****°**
	**of responders (%)**
Infancy (birth to 2 years) / Childhood (3–12 years)	3 (4, 8)
Adolescence (13–20 years)	6 (9, 7)
Early adulthood (21–40 years)	24 (38, 7)
Late adulthood (>40 years)	29 (46, 8)
**Body distribution**	
Dystonia, not better specified	1 (1, 61)
Focal Dystonia	39 (62, 9)
Generalized Dystonia	6 (9, 68)
Hemidystonia	1 (1, 61)
Multifocal Dystonia	6 (9, 68)
Segmental Dystonia	9 (14, 52)
**Diagnosis**	
Acquired (Post-Traumatic-Brain injury) Dystonia	1 (1, 6)
Blepharospasm	1 (1, 6)
Cervical Dystonia	31 (50)
Depression	1 (1,6)
Dopamine responsive Dystonia	1 (1, 6)
Dystonia - parkinsonism	1 (1,6)
Dystonia, not better specified	1 (1, 6)
Focal dystonia, not better specified	1 (1, 6)
Generalized dystonia, not better specified	2 (3, 2)
Idiopathic dystonia, not better specified	1 (1,6)
Idiopathic torsion Dystonia	1 (1, 6)
Idiopathic focal Dystonia	1 (1,6)
Late-onset generalized Dystonia	1 (1, 6)
Meige syndrome	2 (3,2)
Multifocal dystonia, not better specified	3 (4,8)
Musician's Dystonia	1 (1, 6)
Primary generalized Dystonia	1 (1, 6)
Segmental dystonia, not better specified	7 (11, 3)
Writer's cramp	2 (3, 2)
Dystonia, not better specified	2 (3, 2)
**Therapies**	
Oral drugs	29 (46, 8)
Botulinum neurotoxin injections	50 (80, 6)
Rehabilitation	19 (30, 6)
Deep brain stimulation	6 (9, 7)
Others	3 (4, 8)
None	2 (3, 2)
Others: osteopathy, acupuncture, psychological support	
**Combined therapies**	
Only one treatment	24 (38, 71)
Two treatments	25 (40, 32)
Three treatments	11 (17, 74)
Four treatments	0 (0)
None	2 (3, 2)

**Figure 1:**
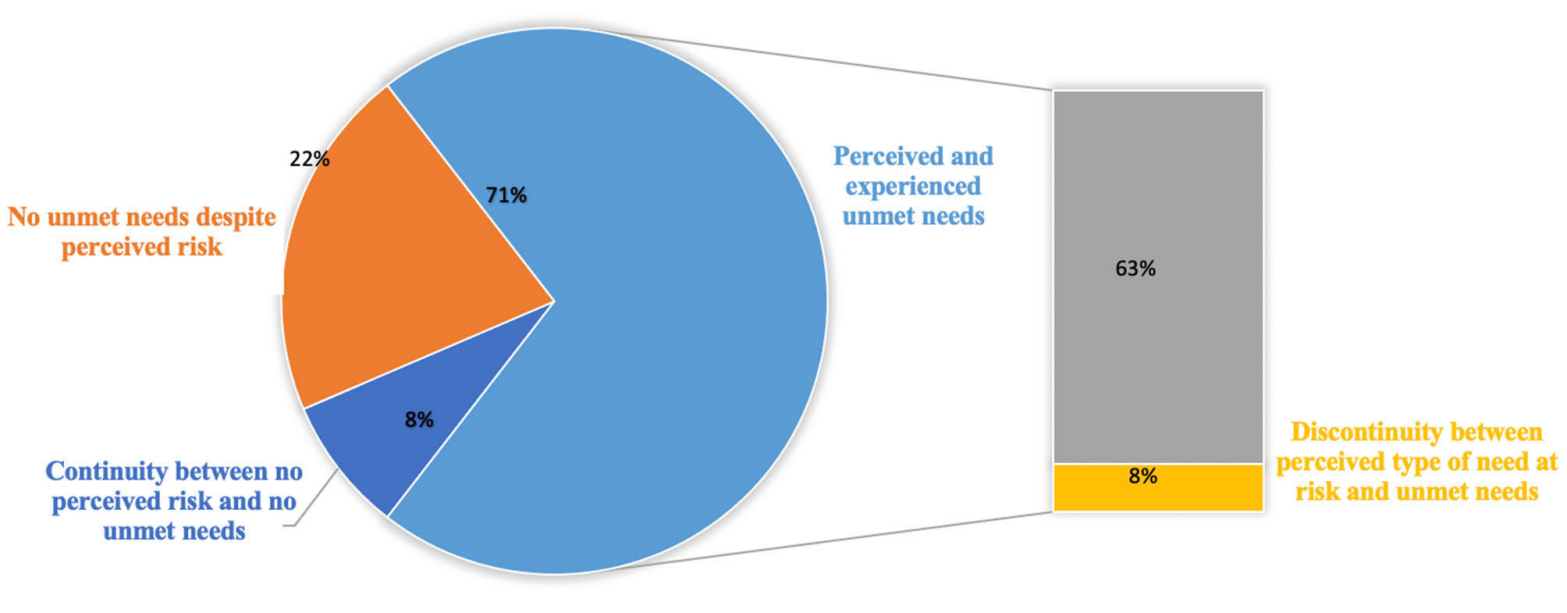
Relationship between perceived and experienced needs during lockdown (*n* = 62).

Figure 1 summarizes how healthcare providers reorganized dystonia-related care in the timeframe of this study and the number of telephone contacts with DPs. Although most centers (75.8%) closed their in-person outpatient clinics, and about half of them (51.06%) even discontinued telephone contact with healthcare providers, data varies across the Italian regions (Figure 1).

### Qualitative Findings

The qualitative analysis yielded six themes summarized in Table 3, with sub-themes and a selection of meaningful quotations from the participants. The main themes are (i) feelings related to COVID-19; (ii) perception of dystonia-related needs; (iii) worsening of symptoms; (iv) work-related changes; (v) relationship with neurologists and services; and (vi) telephonic support.

**Table 3:** Themes, sub-themes and meaningful quotations.

**Theme**	**Sub-theme**	**Meaningful quotations**
***Feelings related to COVID-19***	Feeling calm/serenity	“*Considering my difficulties, I am not afraid of COVID-19.”* (P13) “*I was worried in a quite rational way; I tried to stay updated on the news and especially in the family we used all possible precautions to avoid being infected. I did not want to enter the house with shoes, and we avoided talking to neighbors and people in the supermarket, especially always wearing masks, even now, for the rest we got through this period well.”* (P40)
	Negative oriented feelings	“*Fragile and exposed. I was worried about adding problems to my discomfort. I also take care of my elderly parents, and this did not allow me a complete isolation”* (P15) “*Of course, fear of contracting the virus, since you can also get infected through things that you touch outside.”* (P18) “*Very worried because I already suffer a lot.”* (P27)
***Perception of dystonia-related needs***	Unmet needs	“*Unable to make toxin. Unable to make an appointment. Afterwards there was the fear of COVID and having been abandoned. The symptoms of dystonia, unfortunately, did not go into quarantine.”* (P22) “*My only thought was to give the toxin injections, while COVID seemed to annihilate all other diseases.”* (P46)
	No unsatisfied needs	“*I was pregnant, I had no unmet needs.”* (P45) “*I had no unmet needs.”* (P53)
	Need for more meetings/interviews	“*For advice on what to do as I could not do the botulinum toxin treatment”*. (P24) “*I would like to be followed not every three months but a monthly visit also for emotional comfort.”* (P39)
***Symptoms worsening***	Increase in involuntary movements	“*I had more contractions, more torsion, more pain, more tremors.”* (P03) “*The tension exacerbated the tremor in both my hands.”* (P21) “*I had more spasms in my neck.”* (P13) “*More tremors and spasms.”* (P39) “*Involuntary movements of the neck increased.”* (P56)
	Dystonia-related malfunctions	“*The difficult gait has worsened.”* (P05) “*I have had a loss of balance along with the pains”*. (P20) “*Difficulties with fine movements such as cooking and brushing my teeth.”* (P21) “*An increase or difficulties in writing and fine motor skills.”* (P28) “*Stress and sedentary life”* (P37) “*Dystonia at foot has increased.”* (P41) “*The tongue did not work.”* (P51)
***Work-related changes***	Switching to smart working	“*I worked in smart working with more time for myself and less stress.”* (P31) “*I worked from home, in smart working.”* (P62)
	Job reduction/loss of work	“*Since mid-March, more than 80% of our employees have been laid off”*. (P34) “*My employment contract expired during the lockdown.”* (P35)
	Increasing workload	“*More tension among colleagues.”* (P21) “*I worked much more (primary care department), including Saturdays and Sundays, non-stop”*. (P42) “*I am a nurse; they transferred me to a COVID ward.”* (P54)
***Relationship with neurologists and services***	Neurologist consultations/visits during the lockdown	“*To get a new appointment for infiltration.”* (P01) “*Because after the last toxin injection I had some discomfort.”* (P04) “*To get information about the operation of the department and to give news about my condition.”* (P15) “*To describe the worsening.”* (P21) “*Because of the pregnancy, how I was and advice on epidural and cord donation.”* (P45) “*For appointment cancellation.”* (P52)
	Perceptions following neurologist consultations/visits	“*They asked me how I was. They reassured me.”* (P04) “*Glad I did the treatment. Luckily, I did.”* (P55) “*I felt well, calm, reassured.”* (P57) “*Well, I felt relieved to have had the treatment.”* (P60)
	Negative feelings due to lack of response by the neurologist	“*I felt abandoned and without help.”* (P30) “*A bit confusing.”* (P40)
***Telematic support***	Feeling benefits of telematic support services	“*I was reassured that dystonia was not making me more susceptible to COVID infection”*. (P01) “*It was useful to be able to confront other people suffering from the same disease.”* (P10) “*Interactive webinars, well done and clear. It helped me to have the support of my physiotherapist who disseminated a video with exercises.”* (P18). “*I did not feel abandoned.”* (P31)
	Low support perception of telematic information channels	“*Little information and support.”* (P17) “*I had complex questions with many variables that could not be exhausted in a telephone interview.”* (P38) “*I did not find any help.”* (P43)

#### Feelings Related to COVID-19

Fifty-four responders shared the emotions they experienced in the first wave of the pandemic. Twenty-seven participants said that they felt relatively calm/serene regarding the possibility of contracting the virus. The remaining half of the participants reported that the COVID-19 crisis triggered many negative feelings. Generally, the overall pandemic situation frightened the DPs, making them feel more vulnerable and exposed. In some cases, the fear of virus exposure was enhanced by cohabitating with other family members or being caregivers of elderly parents.

Fourteen participants mentioned that the lockdown was the cause of increased stress, nervousness, fear of suffering more pain within a general emotive context of isolation, and a sense of uncertainty.

#### Perception of Dystonia-Related Needs

From our study, it was revealed that dystonia-related needs were not met during the lockdown. Our respondents reported their concern with the suspension of BoNT, DBS follow-up, and postponement of rehabilitation sessions. They perceived that COVID-19 concentrated healthcare services on the pandemic and neglected treatment for many chronic diseases, including dystonia. In 44 answers, there was an overlap between perceived unmet needs and unsatisfied needs. They were concerned about delays of visits for treatment (i.e., BoNT), physiotherapy sessions, diagnosis results, and psychological support.

However, some participants (*n* = 13) answered that none of their clinical needs were left unsatisfied, even if the risk of unmet needs was reported during the lockdown. Five responses showed continuity between no perceived risk and no unmet need.

Finally, some respondents (*n* = 11) expressed that they wished for more frequent meetings/interviews with the neurologist than they had. They would have preferred to discuss alternatives to BoNT, the worsening of their symptoms, or even to have a chat for psychological relief.

#### Worsening of Symptoms

Twenty-seven responders reported a worsening of symptoms during the lockdown. Many participants (n=21) reported increased involuntary movements, such as tremors or abnormal postures in the form of spasms, tension, and pain. Some participants (n=6) described a dystonia-related condition worsening in daily life, particularly in walking, loss of balance, worsening of problems in the leg and foot, and tongue malfunction. In ten cases, there were significant interferences in daily life and professional functioning, with a consequent increase in sedentariness and a sense of fatigue. Seven participants described how the worsening of symptoms impacted everyday communication like writing and pronouncing words.

#### Work-Related Changes

Of the 62 respondents, 37 complained of work-related changes. Participants reported that they had to switch to smart working, that they could not complete their work, or that their work had been suspended due to their vulnerable condition. Many DPs faced job reduction or non-renewed contracts. Some participants working in the healthcare sector (*n* = 4) complained of increased workload and more tension among colleagues.

#### Appointment With Neurologists and Services

During the lockdown, due to isolation measures for contagion prevention, the patients' appointments with the neurologists/services changed in schedule, timing, and frequency. As mentioned earlier, 11 respondents desired more frequent meetings/interviews with their neurologist. In 33 cases, respondents had to contact their neurologist during the lockdown. A majority could reach the healthcare provider by phone or email (*n* = 22). Participants needed to get in touch with their neurologist for the following reasons: to take/shift/cancel an appointment for botulinum toxin infiltration, to talk about complications caused by BT treatment, to inform the doctor about their state of health, to inform the doctor about the worsening symptoms, to ask for advice, to obtain information about service availability and its schedule, and to seek clarifications regarding cancellation of visits/therapy. Nineteen responders reported that they could visit an outpatient clinic. Some respondents reported that they felt reassured and comforted. They felt “fortunate” to receive the treatment (i.e., BoNT). However, some participants (*n* = 6) said that they received no response from the center/their neurologist, which heightened their perception of being abandoned and confused.

#### Telephonic Support

Some respondents (*n* = 26) reported looking for information by calling their center, contacting the Italian patient association helpline, or connecting to dedicated social network groups. Some participants attended online webinars (*n* = 17). The nature of information the respondents sought was: assurance that dystonia was not a risk factor for getting infected by SARS-CoV-2, dissemination of physical exercises through online videos by physiotherapists, and getting psychological support. The participants who met in online groups described how they felt understood in these groups. Patients found the information and support provided over the telephone helpful and reassuring. While most received the required information, eight DPs did not get any help, and one respondent reported finding little information and support on social networks.

## Discussion

This study is the first attempt at representing the point of view of patients with dystonia, focusing on their unmet needs and adopting qualitative research, which is not common in the movement disorder field. The literature review shows only three studies (31–33) with this approach to dystonia.

The present survey highlights the emotional status of people affected by dystonia: both the pandemic and pandemic-induced impositions contributed to evoking or increasing insecurities. Social isolation came up as a relevant stressor; interestingly, the chance of being infected did not have a similar impact.

A significant finding was the feeling of abandonment while facing dystonia-related issues as COVID-19 attracted all the attention. Participants even used the terms “*to annihilate*” and “*abandoned.”* Indeed, pandemic-induced reorganization of the healthcare system involved physicians, nurses, occupational therapists, and physiotherapists. The risk of many dystonia-related needs not being fulfilled during the first wave of the pandemic matched respondents' experience, as reported, i.e., discontinuation of the botulinum neurotoxin injection or physiotherapy session. In few cases, unsatisfied needs did overlap with perceived unmet needs.

The perceptions of patients with dystonia and pandemic-induced reprioritization of the health system coincided with the rebound of motor and non-motor symptoms. Since dystonia-related symptoms involved involuntary movements, pain, and fatigue, resulting in an impairment of daily functioning, the non-motor symptoms require regularly scheduled visits with the healthcare professionals (9, 34). The consistency of scheduled follow-up plays an essential role in the quality of life and treatment outcome for patients with dystonia. This prompts DPs to seek help and support. Despite the diffuse in-person outpatient clinics being temporarily shut down, respondents experienced reduced contact rates *via* telephone or email with their healthcare providers more so than before the pandemic. This is probably because some hospitals were dedicated exclusively to COVID-19 management, while other physicians and nurses were reassigned to COVID-19 departments. Being able to contact their neurologists provided positive reassurance to patients. Moreover, respondents also sought information about a possible interaction between dystonia and SARS-CoV-2 for psychological support and self-administrable physical exercises *via* different means (i.e., social networks groups, Italian patient association, and phone consultation with a healthcare provider).

Nonetheless, in some cases, the respondents complained that neither the telemedicine-based support nor online initiatives were sufficient, both requiring contact with the professionals.

In this regard, the state of telemedicine before the outbreak of COVID-19 needs mention. Until then, telehealth initiatives were organized locally within a disease-specific program (35, 36) due to several issues, such as the lack of homogenous infrastructures and application standards or inadequate reimbursement for telemedicine services (35, 37). So, without a nationwide telehealth system, the pre-existing limited telemedicine infrastructure was deployed during the first wave of the pandemic (36), in addition to the conventional phone calls or emails to the patients.

Moreover, the work-life balance was also impacted: patients with dystonia working within the healthcare system experienced increased workload; many others had to adapt to smart working in their job or even faced dismissals.

There are different reasons for the recurrence of motor symptoms during the pandemic: First, the interaction between motor and non-motor symptoms (2). Second, the discontinuation of treatment had obvious deleterious repercussions. Recently, Dressler and Adib Saberi explored the effects of BoNT discontinuation during the pandemic-enforced lockdown. More than half of enrolled patients had dystonia, and most complained of a recrudescence of pain and spasms (38). Interestingly, in another study, the worsening of dystonia-related symptoms due to BoNT discontinuation was not linked to the deterioration in the quality of life (12). Also, growing literature supports physiotherapy as an adjuvant therapy (39) that needs regularly scheduled sessions. In the case of device-aided treatments, pandemic countermeasures fostered the implementation of telemedicine in the Initial Deep Brain Stimulation programming (40) for DPs, but it became an additional stressor (13, 32). Third, when patients took advantage of device-aid meetings, uncontrolled motor symptoms aggravated supplementary interference with interpersonal communication, such as in spasmodic dysphonia [“*The tongue did not work.”* (P51)] (33), or social stigma deriving from abnormal postures or involuntary movements (11, 31, 41). Importantly, pain in dystonia has a detrimental impact on occupational status (11), contributing to readjustments in job or dismissal.

This survey underlines how pandemic-related conditions trigger trauma in DPs. In agreement with these findings, a high rate of outpatient clinic shut downs and non-motor symptoms impairment (i.e., mood disorders) was confirmed in DBS-implanted patients (13). To date, fatigue, pain, anxiety, and depression overtake motor symptoms as determinants of QoL and disability (7, 8, 10). Notably, these non-motor symptoms are more prevalent in the general population (7, 10, 11) than those currently acknowledged or diagnosed.

Despite increasing requests, the number of contacts with the local healthcare providers was reduced, resulting in the perception among DPs that they were not being satisfactorily supported. In keeping with these findings, in a recent German study on botulinum neurotoxin, most patients (98%) felt that their rights were not respected (38).

### Strengths and Limitations

The limitation of a convenient sampling method (27) and, if compared to quantitative research, the small number of participants affected the statistical representativeness of the findings and the possibility of correlating perceptions to the sample's characteristics. However, as a qualitative study, it comprises a heterogeneous range of dystonic syndromes and various body distributions with different treatment strategies. Besides, the recruitment method may have contributed to a selection bias. Only the most motivated DPs familiar with seeking online support or using social media may have responded to the survey.

Nonetheless, these study characteristics could be advantageous since they depict a real-life scenario. The proportion of dystonic syndromes is in line with epidemiologic data (2, 3, 11, 42, 43). However, the issues mentioned above recurred and have also been reported elsewhere (38).

As to data collection, given this study's qualitative nature, collecting interview data was preferable to online data collection. Nonetheless, the present survey was designed with close- and open-ended questions that, while limiting the extent of comprehensive explanations from participants, allowed us to gather a rich amount of information from a wide sample of respondents (25).

Moreover, respondents' origin did not cover the entire country, and its representation was non-homogenously arranged. Notwithstanding, as previously mentioned, this study focused on the first wave, particularly the red zones in Italy, which are the declared origins of half of the participants.

## Conclusions

This survey provides evidence of the detrimental condition experienced by DPs during the first wave of the pandemic. Hopefully, these findings might bring DPs' voices to a larger audience, increasing awareness of dystonia as a rare disease. Indeed, even if a first attempt to manage healthcare during emergencies may result in a similar approach to every condition, a more appropriate strategy might include preserving specific treatments, such as BoNT sessions, and expanding telemedicine leveraging electronic devices routinely used by physicians, i.e., DBS-related technology. These theoretical implementations may have a pragmatical impact on healthcare provision in the next steps of this or future pandemics.

## Data Availability Statement

The raw data supporting the results of this article are available upon specific and reasonable request.

## Author Contributions

VR, MD, LG, and FV: research project – conceptualization. VR, MD, FC, FA, SM, LG, and FV: organization and execution and writing—review. VR, MD, and LG: data analysis and writing—first draft. All authors contributed to the article and approved the submitted version.

## Conflict of Interest

The authors declare that the research was conducted in the absence of any commercial or financial relationships that could be construed as a potential conflict of interest.

## Publisher's Note

All claims expressed in this article are solely those of the authors and do not necessarily represent those of their affiliated organizations, or those of the publisher, the editors and the reviewers. Any product that may be evaluated in this article, or claim that may be made by its manufacturer, is not guaranteed or endorsed by the publisher.
